# Extremely Low-Frequency Electromagnetic Fields Affect Myogenic Processes in C2C12 Myoblasts: Role of Gap-Junction-Mediated Intercellular Communication

**DOI:** 10.1155/2017/2460215

**Published:** 2017-05-21

**Authors:** Caterina Morabito, Nathalie Steimberg, Francesca Rovetta, Jennifer Boniotti, Simone Guarnieri, Giovanna Mazzoleni, Maria A. Mariggiò

**Affiliations:** ^1^Department of Neuroscience, Imaging and Clinical Sciences, Unit of Functional Biotechnology and StemTeCh Group, Centro Scienze dell' Invecchiamento e Medicina Traslazionale (CeSI-MeT), “G. d'Annunzio” University of Chieti-Pescara, Chieti, Italy; ^2^Tissue Engineering Unit, Anatomy and Physiopathology Division, Department of Clinical and Experimental Sciences, School of Medicine, University of Brescia, Brescia, Italy

## Abstract

Extremely low-frequency electromagnetic fields (ELF-EMFs) can interact with biological systems. Although they are successfully used as therapeutic agents in physiatrics and rehabilitative practice, they might represent environmental pollutants and pose a risk to human health. Due to the lack of evidence of their mechanism of action, the effects of ELF-EMFs on differentiation processes in skeletal muscle were investigated. C2C12 myoblasts were exposed to ELF-EMFs generated by a solenoid. The effects of ELF-EMFs on cell viability and on growth and differentiation rates were studied using colorimetric and vital dye assays, cytomorphology, and molecular analysis of MyoD and myogenin expression, respectively. The establishment of functional gap junctions was investigated analyzing connexin 43 expression levels and measuring cell permeability, using microinjection/dye-transfer assays. The ELF-EMFs did not affect C2C12 myoblast viability or proliferation rate. Conversely, at ELF-EMF intensity in the mT range, the myogenic process was accelerated, through increased expression of MyoD, myogenin, and connexin 43. The increase in gap-junction function suggests promoting cell fusion and myotube differentiation. These data provide the first evidence of the mechanism through which ELF-EMFs may provide therapeutic benefits and can resolve, at least in part, some conditions of muscle dysfunction.

## 1. Introduction

Modern industrial society produces considerable levels of electromagnetic fields (EMFs), caused by widespread use of electric-power-generating facilities that deliver energy to domestic and industrial appliances. These EMFs are characterized by frequencies of 50 Hz to 60 Hz and are part of the extremely low-frequency (ELF) electromagnetic spectrum [1].

Even if ELF-EMFs are nonionizing radiation, they can interact with biological matter [2]. Since the 1980s, chronic exposure to ELF-EMFs has been implicated in carcinogenesis [3, 4], even if epidemiological studies have not provided conclusive or statistically adequate data. This has mainly been due to confounding by variable exposure times and misclassification selection bias [5, 6].

On the other hand, ELF-EMF-based therapies are commonly used in different medical fields, such as physiatrics and patient rehabilitation [7–9], with apparent beneficial effects. However, at present, the cellular mechanism(s) behind these effects remains unclear. Consequently, experimental investigations addressing how, and to what extent, ELF-EMFs influence living matter become urgent.

Our studies have been specifically designed to provide new insight into the mechanism(s) responsible for the biological effects of ELF-EMFs on skeletal muscle. We previously described the effects of ELF-EMFs on the C2C12 myoblast cell line [10], a well-known in vitro model of skeletal muscle phenotype [11]. We demonstrated that ELF-EMFs affect intracellular reactive oxygen species production, mitochondrial membrane potential, and Ca^2+^ handling, which have been shown to be all patterns strictly dependent on the cell differentiation stage in muscle tissue [12]. Such ELF-EMF-induced changes in mitochondrial activity and Ca^2+^ homeostasis support the hypothesis that exposure to ELF-EMFs can effectively modulate regulation of the myogenesis in C2C12 myoblasts.

Skeletal myogenesis is required for growth, maintenance, and repair of injured muscle [13]. It is a multistep developmental process determined by a complex cascade of events involving development and differentiation of myoblasts, their fusion to form primary and secondary myotubes, and their subsequent maturation into fully developed adult muscle fibers [14, 15].

In recent years, several studies have led to significant improvements in the definition and understanding of the phases of the commitment and differentiation processes of skeletal muscle cells [15–19]. Many in vitro studies have focused on the role of gap-junction-mediated intercellular communication (GJIC) in specific and critical stages of myogenesis [20–23]. It has been hypothesized that GJIC might be involved in the onset of the differentiation process, to coordinate the myoblasts that are committed to differentiate, thus promoting their alignment and fusion, and, consequently, the intercellular diffusion of critical signaling molecules (i.e., Ca^2+^, inositol 1,4,5-trisphosphate, and adenosine 5′-triphosphate) [21, 24, 25]. The evidence that short exposure to ELF-EMFs has effects on both C2C12 myoblasts and myotubes through modulation of their redox status and Ca^2+^ handling [10] also suggests a possible involvement of GJIC and, as a consequence, of long-term biological processes, like myogenesis [26].

To further clarify the mechanism(s) responsible for ELF-EMF-induced effects on myogenesis, in the present study we exposed differentiating C2C12 myoblasts to ELF-EMFs, and, in particular, we monitored their gap-junction permeability, which is considered to be a crucial function for myotube formation during progression through myogenesis.

## 2. Materials and Methods

### 2.1. Equipment

A horizontal solenoid (Oersted Technology Corporation) was designed and built to deliver variable, homogeneous, sine-wave alternating-current magnetic fields at 50 Hz frequency and with intensities from 0.1 mT to 1.0 mT (±2%). This was used to expose large numbers of cells to ELF-EMFs simultaneously [10, 27, 28]. The horizontal cylindrical solenoid presents the following features: length, 340 mm; diameter, 113 mm; number of wire turns, 144; resistance at 20°C (Ω), 0.3; this device was mounted on a nonmagnetic supporting base and powered using a power supply (CW-801P; Elgar Electronics). In particular, the power supply was operated in its “closed-loop current mode”: the output current and the generated magnetic field were consequently regulated against thermal and physical variations/drift of the coil. The final measured value of the solenoid coil constant was 4.830 Gauss/Amp. The solenoid was used in a cell culture incubator (5% CO_2_, 37°C; HERAcell, Kendro Laboratory Products GmbH, Hanau, Germany) for long periods, providing continuous exposure of the cells (1–7 days). During these treatments, any temperature increase in the incubator due to the solenoid was negligible. The tested value of background electromagnetic fields was less than 1 *μ*T (50 Hz). In particular it was in the order of 0.7 *μ*T (50 Hz) in the incubator and outside the switched on solenoid and of 100 nT (50 Hz) outside or inside the switched off solenoid placed in the incubator. Moreover, in laboratory areas between incubators, worktops, and cell cultures hood, electromagnetic fields measured 40–140 nT (50 Hz). The EMF meters used to measure the values of electromagnetic fields are F.W.BELL Tesla meters mod. 4190 (measuring range: 0.01–200 *μ*T, resolution: 0.01 *μ*T) and mod. 6010 equipped with an axial probe mod. HAD61-2508-05T (measuring range: 0.3–300 mT, minimum resolution: 0.01 mT) both from Sypris Test & Measurement (Orlando, FL). The cells were cultured in plastic dishes that are transparent to the ELF magnetic field.

### 2.2. Chemicals and Materials

Unless otherwise indicated, the cell culture media, sera, and antibiotics were from Thermofisher (Monza, Italy), the cell culture plasticware was from Becton Dickinson Falcon (Sacco Srl, Cadorago, Italy), and the reagents and standards were from Sigma-Aldrich (Milan, Italy).

### 2.3. Cell Culture

Undifferentiated C2C12 cells (myoblasts; CRL 1772; American Type Culture Collection, Manassas, VA, USA) were maintained routinely in growth medium (Dulbecco's modified Eagle's medium, 20% fetal bovine serum, 4 mM L-glutamine, 100 IU/mL penicillin, and 100 *μ*g/mL streptomycin). The differentiated phenotype of these C2C12 myoblasts (i.e., myotubes) was obtained by culturing these cells in differentiation medium (Dulbecco's modified Eagle's medium, 2% heat-inactivated horse serum, 4 mM L-glutamine, 100 IU/mL penicillin, and 100 *μ*g/mL streptomycin) for 7 days to 10 days. Both undifferentiated and differentiating C2C12 myoblasts were routinely maintained at 37°C in a humidified 5% CO_2_ atmosphere.

### 2.4. Viability and Proliferation Assays

The analysis of cell viability was performed using trypan blue exclusion tests and cell growth using colorimetric assays based on the reduction of 3-(4,5-dimethylthiazol-2-yl)-2,5-diphenyltetrazolium bromide (MTT). Briefly, C2C12 myoblasts (4.0 × 10^3^ cells/cm^2^) were plated into 96-well plates (Corning-Costar, Milan, Italy) in growth medium. At selected times with the absence and presence of the ELF-EMF treatments, MTT was added to each well to a final concentration of 0.5 mg/mL. After 3 h at 37°C, the plates were centrifuged at 500 ×g for 5 min. The supernatants were removed, and 200 *μ*L dimethyl sulfoxide was added to each well. After 30 min at 37°C, the absorbance of each well was determined using a microplate reader (SpectraMAX 190), at 560 nm. The trypan blue exclusion assay was performed by staining C2C12 myoblasts with a trypan blue dye solution (0.5% in phosphate-buffered saline), and the stained cells were counted using a hemocytometer (Bürker chamber). Blue stained cells were considered nonviable.

### 2.5. Fusion Index

The extent of successful differentiation of C2C12 myoblasts into myotubes was determined by morphological analysis and Hoechst staining of the nuclei [29]. C2C12 myoblasts (4.0 × 10^3^ cells/cm^2^) were seeded in growth medium onto sterile coverslips (Ø, 12 mm) in 24-well plates. After 3 days, the growth medium was shifted to differentiation medium, and the C2C12 myoblasts were induced to differentiate for up to 7 days in the absence and presence of the ELF-EMF treatments. At each time point, the cells were washed twice with phosphate-buffered saline, fixed in 90% ethanol, and stained with Hoechst solution. Images were acquired using an inverted microscope (Olympus IX-70) equipped with a digital camera (Camedia C-5050; Olympus). At least 20 fields were analyzed for each experimental condition. The Fusion Index was quantified as the percentage of Hoechst-stained nuclei located within multinucleated cells (with at least two nuclei, as a result of myoblast fusion), based on the total analyzed nuclei.

### 2.6. Western Blotting

C2C12 myoblasts (4.0 × 10^3^ cells/cm^2^) were seeded in growth medium into 100 mm diameter Petri dishes. After 3 days, the growth medium was shifted to differentiation medium, and the cells were maintained in the absence and presence of the ELF-EMF treatments. At each experimental point, the cells were scraped, lysed, and collected in sample buffer (62.5 mM Tris-HCl, pH 6.8, 2% SDS, 10% glycerol, 0.1 M dithiothreitol, and 0.002% bromophenol blue). Protein concentrations were determined using the protein assay kits (Bio-Rad DC; Bio-Rad, Segrate, Italy). The whole cell extracts were separated using SDS-PAGE on 7.5% or 12.5% (w/v) homogeneous slab gels (40 *μ*g protein/lane) and then transferred onto polyvinylidene fluoride membranes (Immobilon-P; Merck-Millipore, Vimodrone, Italy). Equal loading of protein samples was monitored using Coomassie blue staining of identical gels run in parallel (0.25% Coomassie blue solution; R 250/G 250 1 : 1; Bio-Rad). Red Ponceau S (Sigma-Aldrich) staining of each membrane was used to monitor the transfer efficiency.

The membranes were hybridized with a mouse monoclonal anti-connexin 43 (cx43) antibody (dilution, 1 : 500; Chemicon International Inc., Temecula, CA, USA), a rabbit polyclonal anti-MyoD antibody (dilution, 1 : 500; Santa Cruz Biotechnology Inc., Santa Cruz, CA, USA), or a mouse monoclonal anti-myogenin antibody (dilution, 1 : 500; Santa Cruz Biotechnology Inc.), followed by reaction with horseradish-peroxidase-conjugated anti-mouse or anti-rabbit IgGs (1 : 10,000; GE Healthcare, Cologno Monzese, Italy). The membranes were then incubated with horseradish-peroxidase conjugated anti-IgG, with the relevant proteins detected using chemiluminescence kits (Pierce EuroClone S.p.A., Pero, Italy) and an image acquisition system (Uvitec, Cambridge, UK). An anti-ERK1/2 antibody (1 : 2000 dilution; Santa Cruz Biotechnology Inc.) was used as the loading control.

### 2.7. Microinjection/Dye-Transfer Assay

The differentiating C2C12 myoblasts were tested for their establishment of functional gap junctions using a microinjection/dye-transfer assay, following the protocol proposed by Mazzoleni et al. [30]. Briefly, C2C12 myoblasts (4.0 ×  10^3^ cells/cm^2^) were seeded in growth medium into 60 mm diameter Petri dishes. After 3 days, the growth medium was shifted to differentiation medium, and the cell cultures were maintained in the absence and presence of the ELF-EMF treatments. For the dye-transfer assay, single cells within a monolayer were microinjected with a 10% (w/v) solution of the gap-junction-permeant fluorescent tracer lucifer yellow CH in 0.33 M lithium chloride. Microinjections were performed using glass capillary needles (Clark Electromedical Instruments, Edenbridge, UK) prepared in an automatic puller (Narishige, Tokyo, Japan). The needles were driven using a micromanipulator (Narishige 5240) linked to a fluorescence microscope (Olympus IMT2-SYFII). The fluorescent dye was injected into single cells under nitrogen pressure, using a microinjector (Eppendorf, Hamburg, Germany). Five minutes after the last injection, the cells were fixed with 4% paraformaldehyde in phosphate-buffered saline, and their dye-transfer was evaluated. The extent of functional GJIC was quantified by counting the number of fluorescent cells surrounding the microinjected cells (i.e., the number of dye-coupled cells/injection). For the precise quantification of functional GJIC in the cultures, at least 25 independent microinjection trials/dish were carried out in three separate dishes for each experimental point. Functional GJIC is expressed as the means ± SEM.

Fluorescence and phase-contrast images of untreated and ELF-EMF-treated monolayers were obtained using the inverted fluorescence microscope (Olympus IMT2-SYFII) equipped with a digital camera (Olympus OM-4 Ti reflex).

### 2.8. Statistical Analyses

Statistical analysis was performed using the Prism 4 software for Windows (GraphPad Software Inc., San Diego, CA, USA). Unless otherwise indicated, all of the data are expressed as means ± SD or ± SEM, as specified in the figure legends. Comparisons were made using *t*-tests.

## 3. Results

### 3.1. Effects of ELF-EMF Exposure on C2C12 Myoblast Proliferation and Differentiation

**Figure 1:**
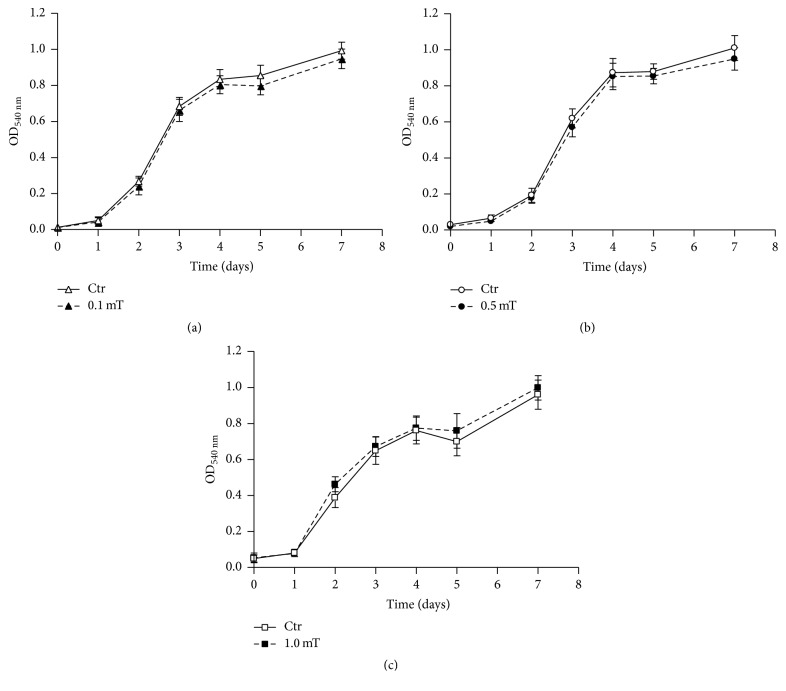
Effects of ELF-EMF treatments on C2C12 myoblast proliferation. Cell proliferation curves derived from MTT colorimetric assays performed on C2C12 myoblasts in the absence (Ctr) and presence of ELF-EMF treatments with 0.1 mT (a), 0.5 mT (b), and 1.0 mT (c). Data are means ± SD from two independent experiments, each performed in six independent culture wells (*n* = 12).

The effects of ELF-EMF exposure on the C2C12 myoblast growth rate and viability were studied using a colorimetric assay and vital dye staining, respectively. These analyses were performed on undifferentiated C2C12 myoblast cultures exposed for up to 7 days to ELF-EMF treatments at different field intensities (0.1, 0.5, and 1.0 mT). As shown in Figure 1, exposure of the C2C12 myoblasts to these ELF-EMFs did not significantly affect either cell proliferation rates or viability; indeed, the numbers of nonviable cells did not differ between the ELF-EMF-treated C2C12 myoblast cultures and their corresponding untreated controls and remained from 10% to 15% of the total cells.

**Figure 2:**
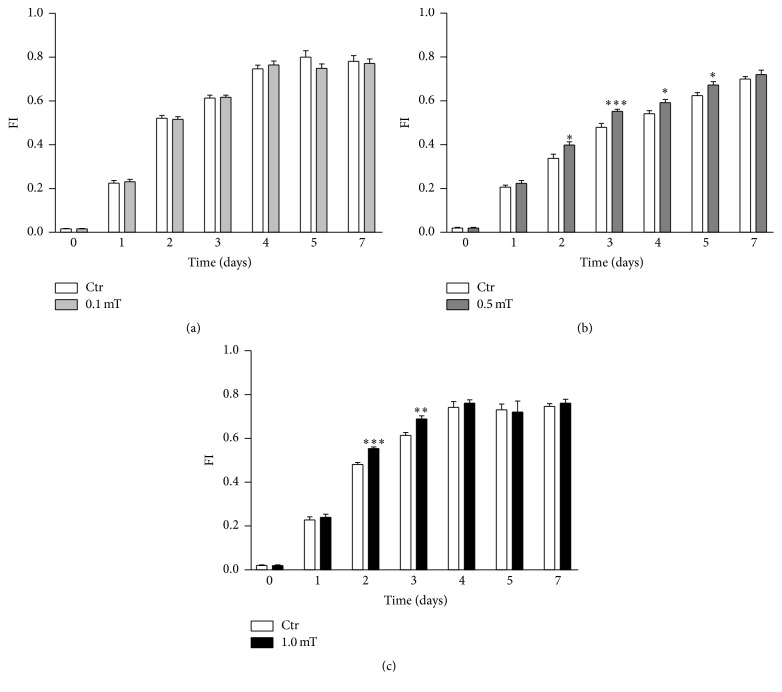
Effects of ELF-EMF treatments on the C2C12 myoblast myogenic process evaluated in the morphological analysis. Percentages of differentiating C2C12 myoblasts expressed as the Fusion Index (see Materials and Methods), as quantified in C2C12 myoblast cultures incubated in the absence (Ctr) and presence of ELF-EMF treatments with 0.1 mT (a), 0.5 mT (b), and 1.0 mT (c). Data are means ± SEM from two independent experiments, each performed in six independent culture wells (*n* = 12). ^*∗*^*p* < 0.05, ^*∗∗*^*p* < 0.01, and ^*∗∗∗*^*p* < 0.001.

The effect of ELF-EMF exposure on the myogenesis process of the C2C12 myoblasts was explored using morphological analysis and quantification of the expression of MyoD and myogenin, two early markers of myogenesis. The morphological analysis was quantified according to the Fusion Index, which was calculated as the percentage of C2C12 myoblasts (of the total) that underwent the differentiation process. The exposure to ELF-EMFs at the 0.5 mT and 1.0 mT intensities induced significant acceleration of the first phases of the myogenic process. Indeed, the C2C12 myoblasts showed significantly higher Fusion Index when exposed to ELF-EMF treatments with 0.5 mT after 2 days to 5 days and with 1.0 mT after 2 days to 3 days (Figures 2(b) and 2(c)). No significant differences were observed for the C2C12 myoblast cultures exposed to ELF-EMFs at 0.1 mT, in comparison with the untreated controls (Figure 2(a)).

**Figure 3:**
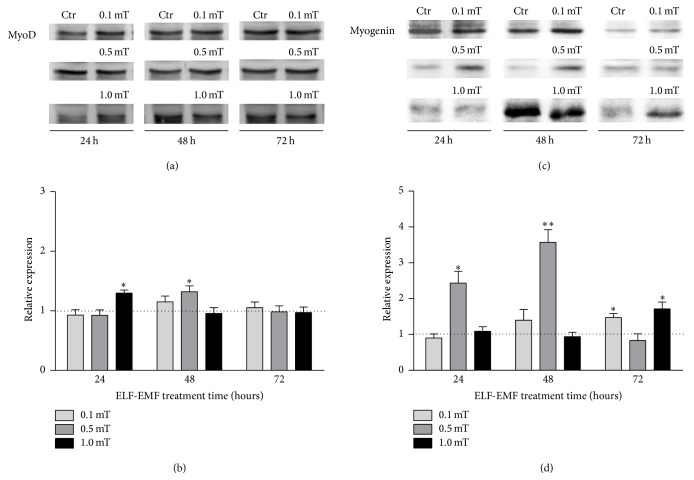
Expression levels of MyoD and myogenin in differentiating C2C12 myoblasts during ELF-EMF treatments. ((a) and (c)) Representative immunoblot of MyoD and myogenin expression levels, respectively. ((b) and (d)) Densitometry analysis of MyoD and myogenin expression levels, respectively, plotted as relative expression calculated by the ratio between OD × mm^2^ of MyoD or myogenin band found in ELF-EMF-treated cells (0.1 mT, 0.5 mT, or 1.0 mT) and OD × mm^2^ of the respective band in the corresponding control (Ctr). Data are means ± SEM from three independent experiments. ^*∗*^*p* < 0.05 and ^*∗∗*^*p* < 0.01 versus corresponding Ctr.

MyoD and myogenin expression levels showed some increases for the C2C12 myoblasts exposed to ELF-EMF treatments, with respect to the corresponding control cells (Figure 3). For the ELF-EMF treatment with 0.1 mT, this increase was evident only for myogenin expression after 72 hours of exposure. The exposure to 0.5 mT intensity triggered an increase of MyoD expression at 48 hours and of myogenin expression at 24 and 48 hours. Differently, 1.0 mT induced increased MyoD expression at 24 hours and increased myogenin expression at 72 hours of exposure.

### 3.2. Influence of ELF-EMFs Treatments on C2C12 Myoblast cx43 Expression and GJIC

The effects of ELF-EMF treatments on differentiating C2C12 myoblasts were also evaluated by an analysis of the efficiency of their gap-junction coupling (dye-transfer efficiency) and by determining their expression levels of cx43, the major gap-junction component in skeletal myoblasts [20, 31, 32].

**Figure 4:**
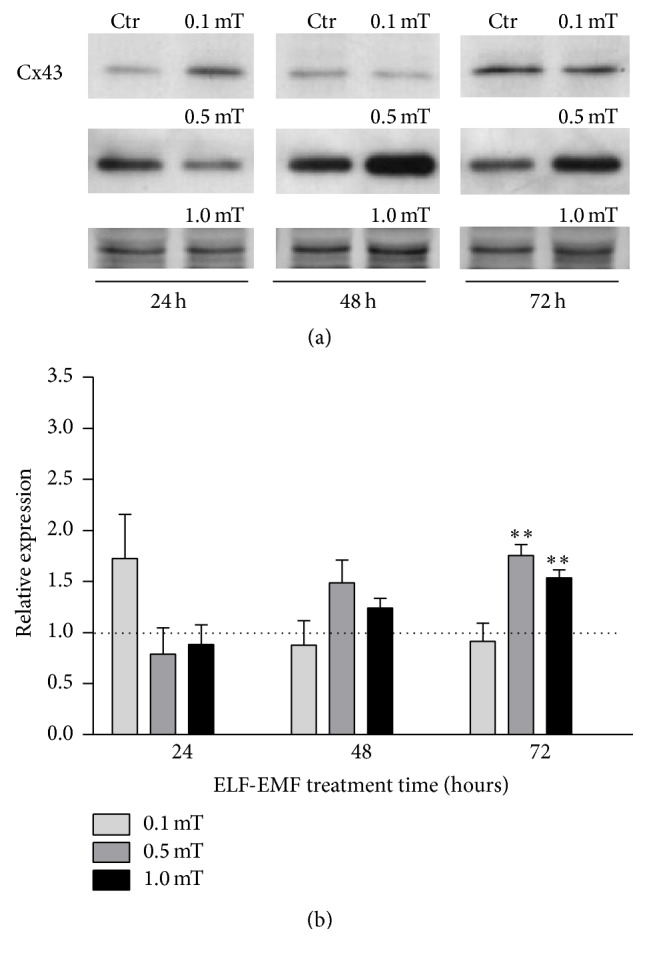
Expression levels of cx43 in differentiating C2C12 myoblasts during ELF-EMF treatments. (a) Representative immunoblot of cx43 expression levels, from three independent experiments. (b) Densitometry analyses plotted as relative expression calculated by the ratio between OD × mm^2^ of the cx43 band found in ELF-EMF-treated cells (0.1 mT, 0.5 mT, and 1.0 mT), and OD × mm^2^ of the respective band in the corresponding control (Ctr). Data are means ± SEM from three independent experiments ^*∗∗*^*p* < 0.01 versus corresponding Ctr.

As illustrated by the representative Western blot and densitometric analyses in Figure 4, during the ELF-EMF treatment with 0.1 mT, a transient, but not significant, increase in cx43 expression was seen only at 24 hours, with respect to the corresponding control cells. Conversely, when the cells were exposed to ELF-EMF treatment with 0.5 mT, or 1.0 mT, these showed an increase in cx43 expression that became significant at 72 hours, with respect to the corresponding control cells (Figure 4).

**Figure 5:**
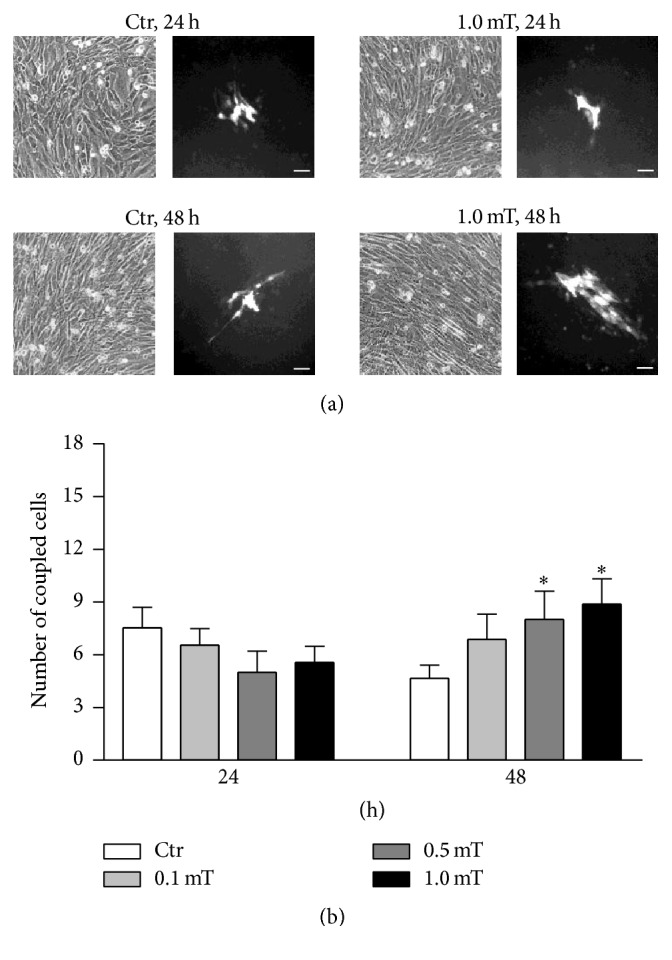
Functional coupling in differentiating C2C12 myoblasts during ELF-EMF treatments. (a) Representative phase contrast and corresponding fluorescence images of the C2C12 myoblasts in the absence (Ctr) and presence of ELF-EMF treatment with 1.0 mT, from the beginning of the differentiation process (*T*0, time 0) and after 24 h and 48 h in differentiation medium. Scale bars, 50 *μ*m. (b) Quantification of dye-coupled cells under the conditions described in (a). Data are means ± SEM from two independent experiments, each performed with at least 10 independent microinjections/plate. ^*∗*^*p* < 0.05 versus corresponding Ctr.

The influence of the ELF-EMF treatment on C2C12 myoblast gap-junction function was tested using the microinjection/dye-transfer assay at the early stages of C2C12 myoblast differentiation (i.e., 24, 48 h). These times were selected on the basis that it is the earlier times that are crucial for the onset of the differentiation program in these C2C12 myoblasts. In addition, after 48 h, the high cell density that was reached by the C2C12 myoblast monolayers made the single-cell approach for the assay difficult. The quantitative analysis of the extent of dye-coupling during the first 48 h of differentiation of the C2C12 myoblasts (e.g., see the representative phase contrast and corresponding fluorescence images in panel (a) and the graph in panel (b) of Figure 5) revealed an increased number of coupled cells in the cultures exposed to ELF-EMF treatment that became significant after 48 h at 1.0 mT, in comparison to the corresponding controls.

The treatment of the differentiating C2C12 myoblasts with ELF-EMFs at 0.5 or 1.0 mT increased not only the expression of the major junction component, cx43, but also its assembly into functional membrane channels, which allowed direct cell-to-cell communication among the cells that were committed to differentiate.

## 4. Discussion

Epidemiological studies on the potential hazards of EMFs for human health have generated many controversies and attracted the attention of the media, the general public, and biomedical researchers [33, 34]. On the other hand, there is evidence of beneficial effects of magnetic fields and EMFs in the treatment of various injuries and diseases [9]. Indeed, clinical benefits for EMFs have been claimed for 20 centuries, and even if there has been perplexity and medical skepticism concerning their use, the application of EMFs with a therapeutic aim is probably one of the most ancient treatments used by Man [35].

Today, both static and time-varying magnetic fields are applied therapeutically with success in the treatment of musculoskeletal injuries or dysfunction [36]. There are also a large number of experimental and clinical studies that have demonstrated that various exogenous EMFs at surprisingly low levels can have effects on a variety of biological systems and processes, most of which are of critical importance for diagnostics and therapy. Along with the evidence from in vitro cellular models, clinical and preclinical studies have suggested that magnetic field and EMF stimulation can accelerate healing processes and influence nerve repair and regeneration [7].

Skeletal muscle also represents a potential target for the biological effect(s) of ELF-EMFs, and this property might be of great importance, due in particular to their diffuse medical applications in physical and rehabilitation medicine. To better clarify this point, the objective of the present study was to investigate the effects of ELF-EMFs on the myogenic process. To achieve this, the C2C12 myoblastic cell line was chosen as a suitable in vitro model of skeletal muscle differentiation. The use of a well-characterized experimental model, the strict control of the experimental procedures, and the optimization of the protocol for this C2C12 myoblast exposure to ELF-EMFs have provided us with improved understanding of the cellular mechanism responsible for ELF-EMF-induced cell modifications, which have remained largely unknown to date.

The data presented here demonstrate that under these experimental conditions ELF-EMFs at 50 Hz and with electromagnetic field strengths from 0.1 to 1.0 mT do not affect either C2C12 myoblast viability or proliferation rate. This thus initially demonstrates that there are no direct toxic effects of ELF-EMFs on this cell model. Even if this finding is in apparent contrast with what has been observed with other cell models, it is important to bear in mind that, in the many studies that have reported that ELF-EMFs can act on cell proliferation and cell-cycle progression, these effects have depended on the cell phenotype, as well as on the intensity, frequency, and wave form of ELF-EMFs applied, and length of the exposure [37–42].

In recent decades, a large amount of evidence has demonstrated prodifferentiating effects of ELF-EMFs on different cell phenotypes, and interesting data have also been obtained using adult stem cells, with the suggestion that ELF-EMFs represent an efficacious therapeutic approach [28, 39, 43–45]. On the other hand, the mechanism(s) of action of ELF-EMFs on physiological processes and their intracellular molecular targets are far from being defined. In the cell model in the present study, the presence of ELF-EMFs enhanced the myogenic process. This process progresses through a highly ordered sequence of events, including morphological changes (i.e., cell alignment, fusion) and specific time-courses of gene expression patterns [46]. In addition, published data have strongly supported a key role for GJIC in the first phase of myogenic differentiation, when myoblasts that are committed to differentiate then adhere and fuse, to form gap junctions. These gap junctions then allow the intercellular diffusion of critical signals, such as Ca^2+^, inositol 1,4,5-trisphosphate, and adenosine 5′-triphosphate [24]. Indeed, when blockers of the permeability of these junction channels are used, or in the presence of inducible deletion of cx43, downregulation of myogenic factors is seen [21–23, 47]. Furthermore, transfection of rhabdomyosarcoma cells with cx43 cDNA was shown to induce cell differentiation [48]. In particular, in myoblasts, cx43 expression is predominant, and it is a prerequisite for their fusion [32]. Indeed, cx43 gap-junction channels have been shown to provide the intercellular signaling pathways that are required for normal timing of skeletal muscle ontogeny and regeneration [20]. In our cell model, ELF-EMFs at 0.1 mT only transiently affected cx43 expression but at 0.5 mT and 1.0 mT modified GJIC activity through increases in both the expression of the cx43 protein and cell coupling in cultures committed to differentiate. These data are complementary to our previous report regarding the evidence that ELF-EMFs can affect the Ca^2+^ handling and redox status of C2C12 myoblasts [10]. These processes are responsible for the phenotypic cell reactions to environmental stimuli, and they might provide the means through which ELF-EMFs can accelerate the myogenesis process through increased extent of the GJIC, as the regulation of Ca^2+^ fluxes by gap junctions and their response to oxidative stress have been well demonstrated [49].

## 5. Conclusions

In conclusion, although our data do not show clear dose-dependence or threshold-induced effects, ELF-EMFs in the mT intensity range accelerated the myogenic process in these C2C12 myoblasts. In the same cell model, ELF-EMFs can affect the cellular redox state and intracellular Ca^2+^ dynamics [10] and, consequently, cell metabolic activity. All of these events can be proposed as key triggers involved in increases in specific myogenic markers (e.g., MyoD and myogenin) and the promotion of cell fusion, which is made more efficient by the increased GJIC activity, all of which are steps that can be enhanced by ELF-EMFs.

Taken together, all these evidences indicate for the first time the mechanism of action through which ELF-EMFs can promote skeletal muscle differentiation. This thus provides a scientific basis to what is considered an efficacious therapeutic means to resolve, at least in part, some conditions of muscle dysfunction.
